# Comparative chloroplast genome analysis of four *Trigonella* species: structural rearrangements, gene loss, and phylogenetic relationships

**DOI:** 10.3389/fpls.2026.1821377

**Published:** 2026-06-11

**Authors:** Ramawatar Nagar, Vandna Patial, Prashant Mohanpuria, Nimmy MS, Sharda Choudhary

**Affiliations:** 1 ICAR-National Institute for Plant Biotechnology, New Delhi, India; 2 Division of Biochemistry, ICAR-IARI, New Delhi, India; 3 School of Agriculture Biotechnology, Punjab Agriculture University, Ludhiana, India; 4 ICAR-National Research Centre on Seed Spices, Ajmer, India

**Keywords:** chloroplast genome, inverted repeat lacking clade, molecular markers, paraphyly, pseudogene, structural rearrangement, *Trigonella*

## Abstract

Fabaceae is one of the most structurally dynamic plant families for chloroplast genome evolution, particularly within the inverted repeat-lacking clade (IRLC), where the loss of one inverted repeat predisposes plastomes to accelerated rearrangement and gene loss. Here, we present the complete chloroplast genomes of four *Trigonella* species, *T. foenum-graecum*, *T. corniculata*, *T. caerulea*, and *T. gladiata*, and perform a comprehensive comparative analysis of their plastome evolution and phylogenetic relationships. All four genomes range from 124,823 to 125,437 bp, each encoding 77 protein-coding genes, 30 tRNA genes, and four rRNA genes. Three genes, *infA*, *rpl22*, and *rps16*, are retained as pseudogenes in one or more species, capturing distinct stages of ongoing plastid-to-nucleus gene transfer. Whole-genome alignment revealed a complex structural rearrangement in the *T. foenum-graecum* plastome, involving the inversion and translocation of four locally collinear blocks across a ~35.5 kb region, a pattern absent from the three collinear species. Most protein-coding genes evolve under strong purifying selection; five genes (*clpP*, *ycf2*, *cemA*, *ycf1*, and *rpl20*) had Ka/Ks > 1, with only *clpP* reaching statistical significance. Nucleotide diversity analysis identified *clpP* as the most variable coding sequence, and the *ycf2*–*trnI*-CAU spacer, *trnR*-UCU–*atpA*, and *rps15*–*ycf1* as the most variable intergenic regions. SSR analysis identified 101 polymorphic loci with associated primer pairs, providing resources for population-level studies. Phylogenetic analysis confirmed that *Trigonella* is not a natural group, with *T. corniculata* resolving as sister to *Melilotus albus* and *M. officinalis* with maximum bootstrap support, providing strong evidence for a formal taxonomic revision of the genus.

## Introduction

The chloroplast is the organelle responsible for photosynthesis in plants and algae. Like mitochondria, chloroplasts contain their own genome, which in most plants takes the form of a circular DNA molecule between 120 and 160 kb in length. The chloroplast genome of most flowering plants follows a well-defined four-part structure, consisting of two copies of an inverted repeat (IR) region that flank a large single-copy (LSC) and a small single-copy (SSC) region (Jansen et al., 2005). This organization is remarkably stable across angiosperms, with most chloroplast genomes encoding around 30 tRNA, four rRNA, and 80 protein-coding genes in a largely conserved order (Daniell et al., 2016; Dobrogojski et al., 2020).

One well-known exception to this pattern is found in the legume subfamily Papilionoideae, where a large group of species has lost one copy of the inverted repeat. This group is known as the inverted repeat-lacking clade, or IRLC (Magee et al., 2010; Wojciechowski et al., 2004). This loss reduces the genome to a bipartite organization and has profound evolutionary consequences, including elevated rates of structural rearrangement, accelerated nucleotide substitution rates in single-copy regions, and the gradual loss or transfer of certain plastid genes to the nucleus (Li et al., 2018; Wang et al., 2018). For these reasons, IRLC members offer a useful window into how chloroplast genome structure shapes molecular evolution over time.

Within the IRLC, the tribe Trifolieae contains several economically important genera, including *Trigonella*, *Melilotus*, and *Medicago*, whose boundaries have long been a source of taxonomic debate. Phylogenetic studies using nuclear and plastid markers have consistently shown that *Trigonella*, as currently defined, is not a natural group, with *Melilotus* falling inside it rather than as a separate genus (Dangi et al., 2016; Steele et al., 2010). Plastome sequences of *Trigonella foenum-graecum* are reported in NCBI GenBank. However, a multi-species comparative analysis spanning the genus within an IRLC framework has not been carried out, leaving important questions about structural evolution, gene loss, and the relationships between *Trigonella* and *Melilotus* unanswered.

In this study, we sequenced and analyzed the complete chloroplast genomes of four *Trigonella* species: *T. foenum-graecum* L., *T. corniculata* (L.) L., *T. caerulea* (L.) Ser. and *T. gladiata* Steven ex M. Bieb. The genomes of *T. foenum-graecum* and *T. corniculata* were sequenced as part of this study, while *T. caerulea* and *T. gladiata* were assembled from publicly available sequencing data. Using these four genomes, we aimed to: (1) describe and compare plastome structure and gene content within *Trigonella*; (2) identify patterns of nucleotide divergence and selection pressure and assess whether any variable regions could serve as molecular markers; (3) test whether whole-plastome phylogenetics clarifies the disputed relationships within *Trigonella* and between *Trigonella* and *Melilotus*.

## Materials and methods

### DNA extraction and sequencing

Seeds of *T*. corniculata cv. Pusa Kasuri Mathi and *T. foenum-graecum* cv. AFG-3 were grown under controlled laboratory conditions. Leaf tissue was collected from the two-week-old seedlings, and genomic DNA was extracted using a modified cetyltrimethylammonium bromide (CTAB) protocol (Nagar and Schwessinger). High-quality genomic DNA was used for library preparation using the NEBNext^®^ Ultra™ II DNA Library Prep Kit (NEB, UK), following the manufacturer’s instructions. Cluster generation and paired-end sequencing were performed on the Illumina NovaSeq 6000 platform using 2 × 150 bp paired-end chemistry, according to the manufacturer’s protocols. The resulting paired-end reads were used for downstream genome assembly and analysis.

To compare the genomes of these two *Trigonella* species with others, the chloroplast genomes of the two other Trigonella species, *T. caerulea* and *T. gladiata*, were assembled using whole-genome sequence data obtained from the NCBI’s Sequence Read Archive (SRA). The raw whole genome sequencing data for *T. gladiata*, *T. caerulea* were retrieved from accession no ERR14010450 (BioProject PRJEB82787) and ERX13449884 (BioProject PRJEB82787), respectively. These data were sequenced and submitted by the French National Sequencing Centre (Genoscope).

### Chloroplast genome assembly and annotation

The quality of the fastq files was assessed using the FastQC tool (https://www.bioinformatics.babraham.ac.uk/projects/fastqc/). To remove the adapter and poor-quality sequence, we used Trim Galore v0.4.0 (https://www.bioinformatics.babraham.ac.uk/projects/trim_galore/). Chloroplast genomes were *de novo* assembled using the CGAS v1.0.1 with the --module 1 command (Abdullah and Tian, 2026). CGAS uses GetOrganelle v1.7.2 (Jin et al., 2020) for assembling the chloroplast genome from short Illumina reads. It also maps the raw reads back to the chloroplast contig using bwa-mem v0.7.19 (Li and Durbin, 2009) to produce a BAM file. We used the BAM file to plot the coverage depth using the bam2plot (Rosenbaum, 2025) for validation (**Supplementary Figures 1A–D**). The chloroplast genome was then annotated with multiple tools specialized for the plastid annotation, such as GeSeq v2.03 (Tillich et al., 2017) and PGA (Qu et al., 2019). For both annotation tools, the *chloroplast sequence of T. foenum-graecum* (NC_042857.1) was used as a reference. The annotations from these were manually checked for CDS size, start codons, and stop codons. A manually created GenBank file was then used for the plotting, a circular map of the chloroplast genome using OGDRAW v1.3.1 (Greiner et al., 2019). The fully annotated chloroplast genome sequences were submitted to the NCBI GenBank database, and accession numbers were obtained: *Trigonella caerulea* (BK075123), *Trigonella gladiata* (BK075124), *T*. *foenum*-*graecum* cv. AFG-3 (PX972516), *T*. *corniculata* cv. Pusa Kasuri Mathi (PX972515).

### Codon usage analysis

Codon usage was analyzed using CodonW v1.4.4 (available at http://codonw.sourceforge.net). For each species, the coding sequences of 77 functional plastid genes were extracted from the annotated GenBank files. CDS sequences were concatenated into species-specific FASTA files and submitted to CodonW for the analysis of relative synonymous codon usage (RSCU and amino acid composition frequency. All analyses were performed using the standard genetic code.

### SSR analysis

The SSRs were detected using the MISA Perl Script v2.1 (Beier et al., 2017) with minimum thresholds of 10, 6, 5, 5, 4, and 4 repeat units for mono-, di-, tri-, tetra-, penta-, and hexanucleotide motifs, respectively. Polymorphic loci were identified by positional matching (±300 bp window) across species, retaining loci showing presence/absence variation or length (repeat number) differences. Primers were designed from flanking sequences targeting a ~150 bp upstream/downstream region, selecting 20-mer primers with GC content closest to 50% and Tm closest to 58°C (Wallace rule).

### Comparative analysis of chloroplast genome structure

For structural analysis of *Trigonella* plastomes, two publicly available chloroplast genome sequences of *T. foenum-graecum* (*NC_042857.1* and *MN736956.1*) were included in the analysis. Before alignment, all chloroplast genomes were reoriented relative to the *rbcL* gene using Rotate v1.0 (Durbin et al., 2023) and subsequently reannotated using GeSeq (Tillich et al., 2017). Whole-genome alignments were performed using the progressiveMauve algorithm, implemented in Mauve, to identify locally collinear blocks (LCBs) (Darling et al., 2004). For sequence conservation analysis and visualization, the chloroplast genomes of four Trigonella species were aligned using Shuffle-LAGAN mode with the chloroplast genome of *T*. *foenum*-*graecum* as a reference and visualized using the mVISTA tool (Brudno et al., 2003).

### Nucleotide diversity analysis

To estimate nucleotide diversity among the four *Trigonella* species, the sequences of all the shared coding genes, along with 78 homologous intergenic sequence blocks across species, were aligned individually using MAFFT v7.525 (Katoh et al., 2002), and nucleotide diversity (π) was calculated using the DnaSP 6 v6.12.03 (Rozas et al., 2017). The π values were estimated following the method of Nei and Li (Nei and Li, 1979), which calculates the mean number of pairwise nucleotide differences per site among all possible sequence pairs. A line graph of nucleotide diversity was plotted using ggplot2 in the R environment.

### Molecular evolution analysis

To investigate the selection pressures acting on chloroplast protein-coding genes, CDS sequences shared across all four *Trigonella* species were extracted from the GenBank annotations, translated into amino acid sequences, and aligned using MAFFT (Katoh et al., 2002). The resulting amino acid alignments were then back-translated into codon-aware nucleotide alignments using PAL2NAL v14.1 (Suyama et al., 2006). Pairwise nonsynonymous (Ka) and synonymous (Ks) substitution rates were then estimated for each gene using KaKs_Calculator v2.0 (Wang et al., 2010), and the Ka/Ks ratio (ω) was calculated as a measure of selective pressure, where values below 1 indicate purifying selection, values equal to 1 indicate neutral evolution, and values above 1 are consistent with positive selection.

### Phylogenetic analysis

For phylogenetic analysis, the four *Trigonella* species, along with 44 taxa, were selected, representing different genera of the IRLC clade and two outside (*Phaseolus vulgaris* and *Phaseolus lunatus*) to root the tree. Shared protein-coding genes from all the species were extracted from GenBank files. A total of 64 orthologous protein-coding genes were identified, and their coding sequences were extracted and concatenated in a multi-FASTA file. The CDS sequences were aligned using MAFFT v7.525 with the auto mode (Katoh et al., 2002). Alignment blocks that were poorly aligned were removed using trimAl v1.5.rev0 with automated trimming settings (Capella-Gutiérrez et al., 2009). The clean alignment file was then used for the maximum-likelihood phylogenetic analysis using RAxML v8.2.12 (raxmlHPC-PTHREADS). The GTRGAMMA nucleotide substitution model was used for the ML analysis with 1,000 bootstrap replicates (Stamatakis, 2014). The tree was visualized using the iTOL web tool (https://itol.embl.de/) (Letunic and Bork, 2016).

## Results

### General features of the *Trigonella* chloroplast genomes

**Figure 1:**
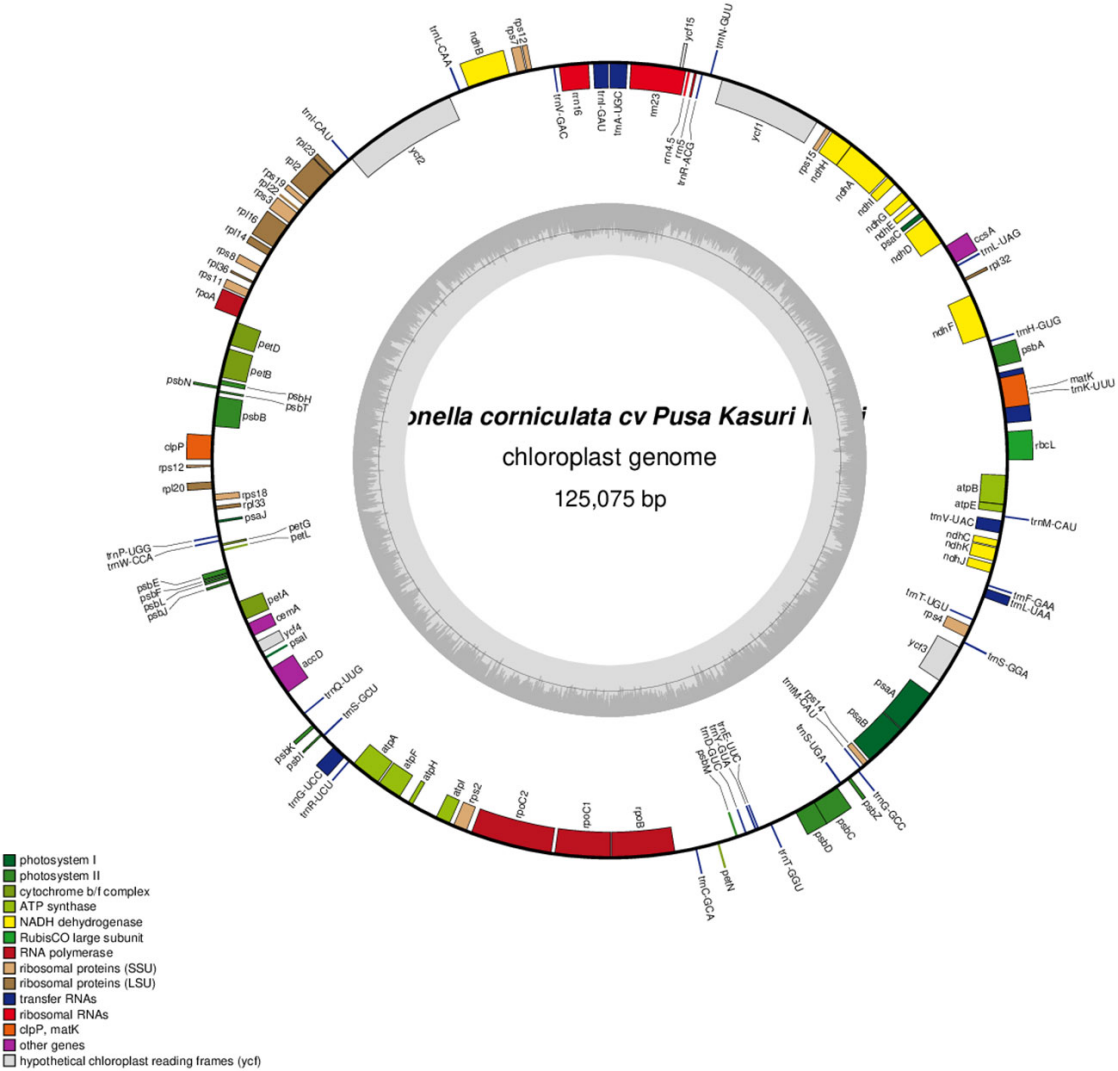
Circular genome maps of the *Trigonella corniculata* chloroplast genome. Genes shown on the outer ring are transcribed clockwise; genes on the inner ring are transcribed anticlockwise. The inner grey ring represents GC content across the genome. Gene arrows above and below the backbone represent genes on the forward (+) and reverse (−) strand, respectively, color-coded by functional category as shown in the legend.

The complete chloroplast genomes of four *Trigonella* species, T. *caerulea*, *T. corniculata*, *T. foenum-graecum*, and *T. gladiata*, were assembled and annotated (**Figure 1** and **Supplementary Figure 2A-C**). All four annotated genomes were submitted to the NCBI GenBank database (**Table 1**). The genome size of all four species was highly conserved, ranging from 124,823-125,437 bp, a typical size reported for members of the inverted repeat lacking clade (IRLC) of the Fabaceae family (**Supplementary Figure 1**). The GC content of the four species was ~34%, reflecting the AT-rich composition typically reported for plastid genomes. Each genome encodes 30 tRNA genes, four rRNA genes, and 77 functional protein-coding genes.

**Table 1:** General features of the four *Trigonella* species chloroplast genomes.

Feature	*T. caerulea* (BK075123)	*T. corniculata* cv. Pusa Kasuri Mathi (PX972515)	*T. foenum-graecum* cv. AFG-3 (PX972516)	*T. gladiata* (BK075124)
Genome size (bp)	125,437	125,075	125,251	124,823
Genome topology	Circular	Circular	Circular	Circular
GC content (%)	33.79	33.71	33.92	33.90
AT content (%)	66.21	66.29	66.08	66.10
Total genes (functional)	111	111	111	111
Protein-coding genes (CDS)	77	77	77	77
tRNA genes	30	30	30	30
rRNA genes	4	4	4	4
Intron-containing genes				
CDS genes with introns	11	11	11	11
*atpF, clpP, ndhA, ndhB, petB, petD, rpl2, rpl16, rpoC1, rps12*; plus, *rps16*ψ in *T*. *caerulea* and *T. gladiata*	*atpF, clpP, ndhA, ndhB, petB, petD, rpl2, rpl16, rpoC1, rps12, rps16*	*atpF, clpP, ndhA, ndhB, petB, petD, rpl2, rpl16, rpoC1, rps12*	*atpF, clpP, ndhA, ndhB, petB, petD, rpl2, rpl16, rpoC1, rps12*	*atpF, clpP, ndhA, ndhB, petB, petD, rpl2, rpl16, rpoC1, rps12, rps16*
Genes with two introns	*ycf3*	*ycf3*	*ycf3*	*ycf3*
tRNA genes with introns	*trnA-UGC, trnG-UCC, trnI-GAU, trnK-UUU, trnL-UAA, trnV-UAC*	*trnA-UGC, trnG-UCC, trnI-GAU, trnK-UUU, trnL-UAA, trnV-UAC*	*trnA-UGC, trnG-UCC, trnI-GAU, trnK-UUU, trnL-UAA, trnV-UAC*	*trnA-UGC, trnG-UCC, trnI-GAU, trnK-UUU, trnL-UAA, trnV-UAC*
Pseudogenes (non-functional gene remnants)				
*infA*	Present (21 amino acid fragment)	Absent	Absent	*Absent*
*rpl22*	Present (40 amino acid fragment)	Present (32 aa fragment)	Absent	Present (40 amino acid fragment)
*rps16*	Present (2 premature stop codons)	Absent	Absent	Present (1 premature stop codon)

An additional three genes, *infA*, *rpl22*, and *rps16*, are variably retained across species as truncated, non-functional remnants (**Table 1**). In *T. caerulea*, the only species retaining an *infA* sequence, the annotated open reading frame encodes just 21 amino acids, less than 30% of the ~72 amino acids required for a functional translation initiation factor 1. Similarly, *rpl22* is present as a severely truncated fragment in *T. caerulea*, *T. corniculata*, and *T. gladiata*, encoding between 32 and 40 amino acids compared to the ~125 amino acids of a functional L22 protein, with the downstream sequence showing multiple stop codons in all reading frames. For *rps16*, premature in-frame stop codons disrupt the reading frame at positions 25 and 44 in *T. caerulea* and at position 36 in *T. gladiata*. These findings confirm that all three genes are pseudogenes in the chloroplast genomes of the four *Trigonella* species.

### Structural organization of *Trigonella* chloroplast genomes

**Figure 2:**
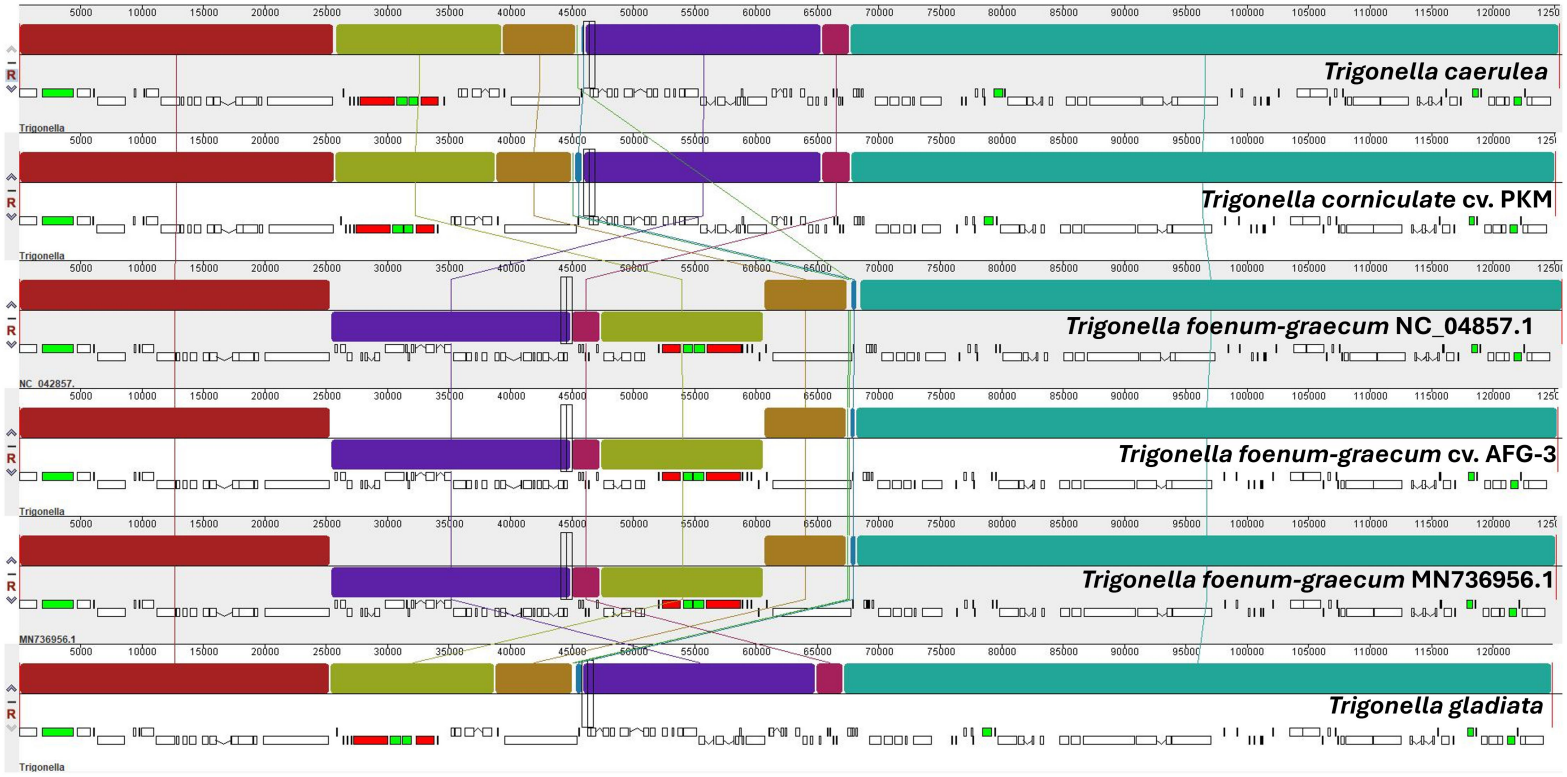
Mauve whole-genome synteny alignment of four *Trigonella* chloroplast genomes. Locally collinear blocks (LCBs) identified by progressive Mauve are displayed as colored rectangles for each genome, with connecting lines indicating homologous blocks across genomes. Blocks above the central axis are in the forward orientation; blocks below are inverted relative to the reference.

**Figure 3:**
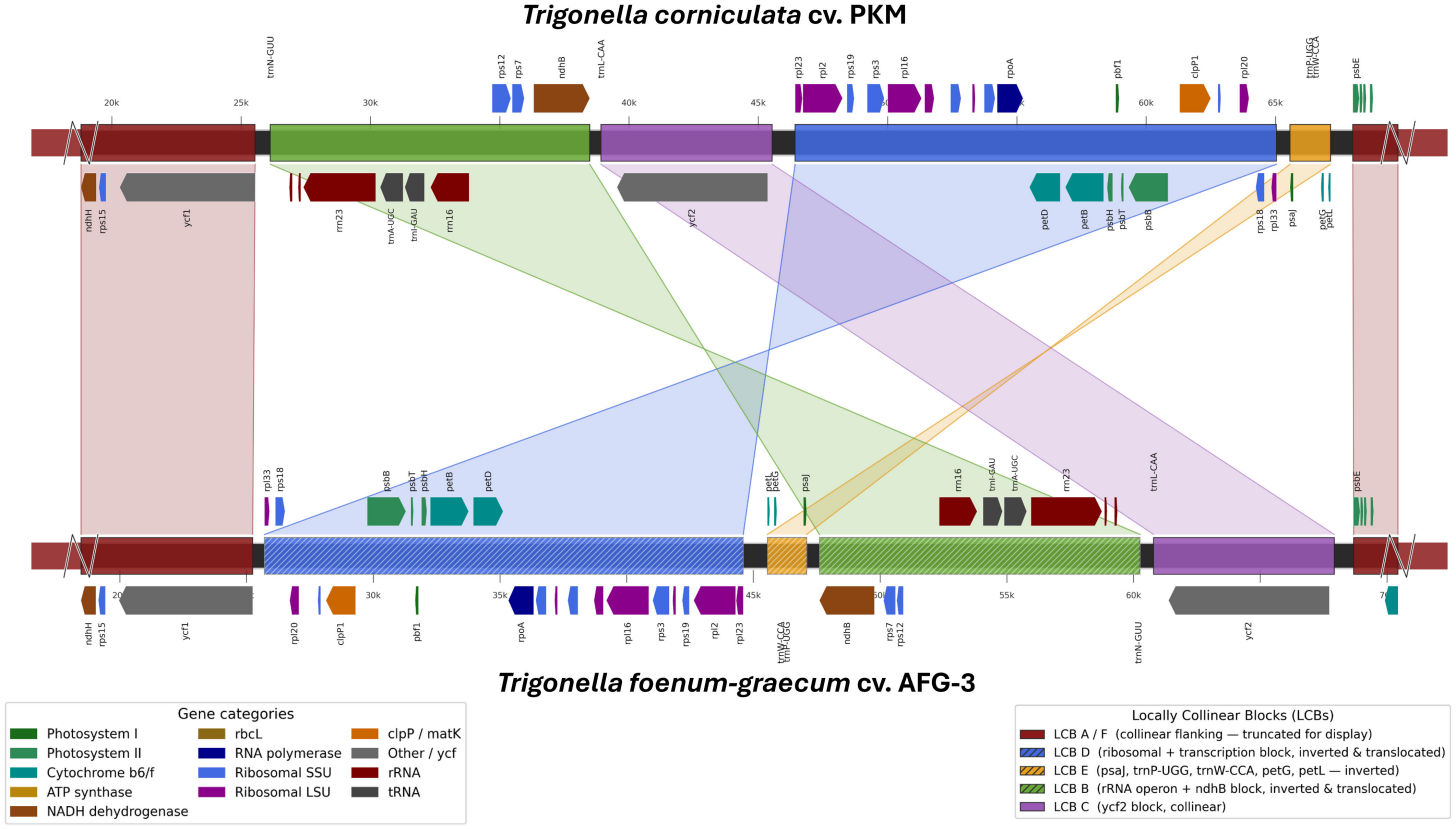
Structural comparison of the *Trigonella corniculata* and *Trigonella foenum-graecum* chloroplast genomes. Linear genome maps are shown for *T. corniculata* cv. PKM (top, 125,075 bp) and *T. foenum-graecum* cv. AFG-3, displaying the rearranged regions. Colored blocks on each genome represent locally collinear blocks (LCBs) identified by whole-genome alignment. Connecting ribbons between the two genome tracks link homologous blocks; crossed ribbons indicate both inversion and translocation. Gene arrows above and below the backbone represent genes on the forward (+) and reverse (−) strand, respectively, color-coded by functional category as shown in the legend.

*T. caerulea*, *T. corniculata*, and *T. gladiata* displayed near-perfect collinearity across all locally collinear blocks (LCBs), whereas *T. foenum-graecum* was the only species showing complex structural rearrangements relative to the other three (**Figure 2**). Four LCBs spanning approximately 35.5 kb were either inverted or translocated in *T. foenum-graecum* compared to the other species (**Figure 3**). Specifically, LCB D, located at positions 46,425–65,041 bp in *T. corniculata*, was found in an inverted orientation at positions 25,709–44,602 bp in *T. foenum-graecum*. Similarly, LCB B, spanning 26,129–38,485 bp in *T. corniculata*, was relocated to positions 47,607–60,259 bp in *T. foenum-graecum*, also in an inverted orientation. A smaller block (LCB E) was likewise inverted and repositioned between LCB D and LCB B in *T. foenum-graecum* (45,554–47,104 bp). In contrast, the *ycf2*-containing block (LCB C), although shifted from its position in *T. corniculata* (38,914–45,541 bp) to a later position in *T. foenum-graecum* (60,793–67,923 bp), retained its original orientation. This pattern of rearrangement is consistent with a series of inversion events likely mediated by illegitimate recombination, a phenomenon commonly observed in inverted repeat-lacking clade (IRLC) plastomes, where the stabilizing influence of the inverted repeat is absent (Maréchal and Brisson, 2010; Palmer and Thompson, 1982).

**Figure 4:**
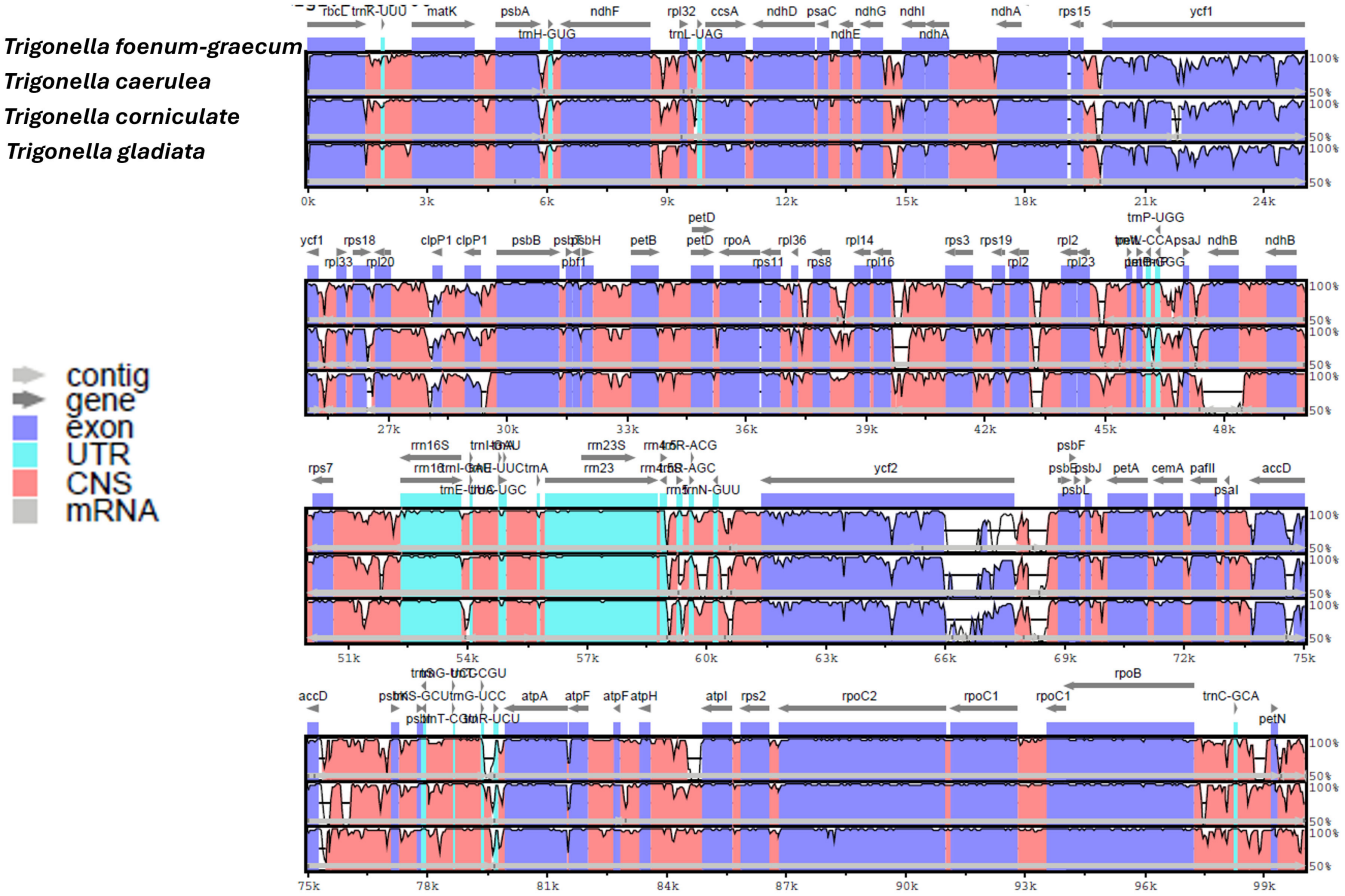
mVISTA sequence identity plot of four *Trigonella* chloroplast genomes. The y-axis represents percentage sequence identity (0–100%). Colored regions indicate conserved elements: blue = exonic (protein-coding) regions; salmon = conserved non-coding sequences (CNS). Gene annotations are displayed above the plots. High peaks correspond to conserved regions, while the valleys correspond to reduced identity.

For sequence conservation analysis, pairwise sequence identity across the four genomes was assessed using mVISTA (**Figure 4**). The mVISTA alignment confirmed broadly high sequence identity across all four genomes, with protein-coding exons, particularly *rbcL*, *matK*, *psbA*, *rpoB/C*, *atpA/B*, and the *rRNA* operon showing the highest conservation. This high level of sequence conservation among the protein-coding genes reflects strong purifying selection on the plastid-encoded proteome. Non-coding intergenic spacer regions showed more pronounced sequence divergence.

### Simple sequence repeat in *Trigonella* chloroplast genomes

**Figure 5:**
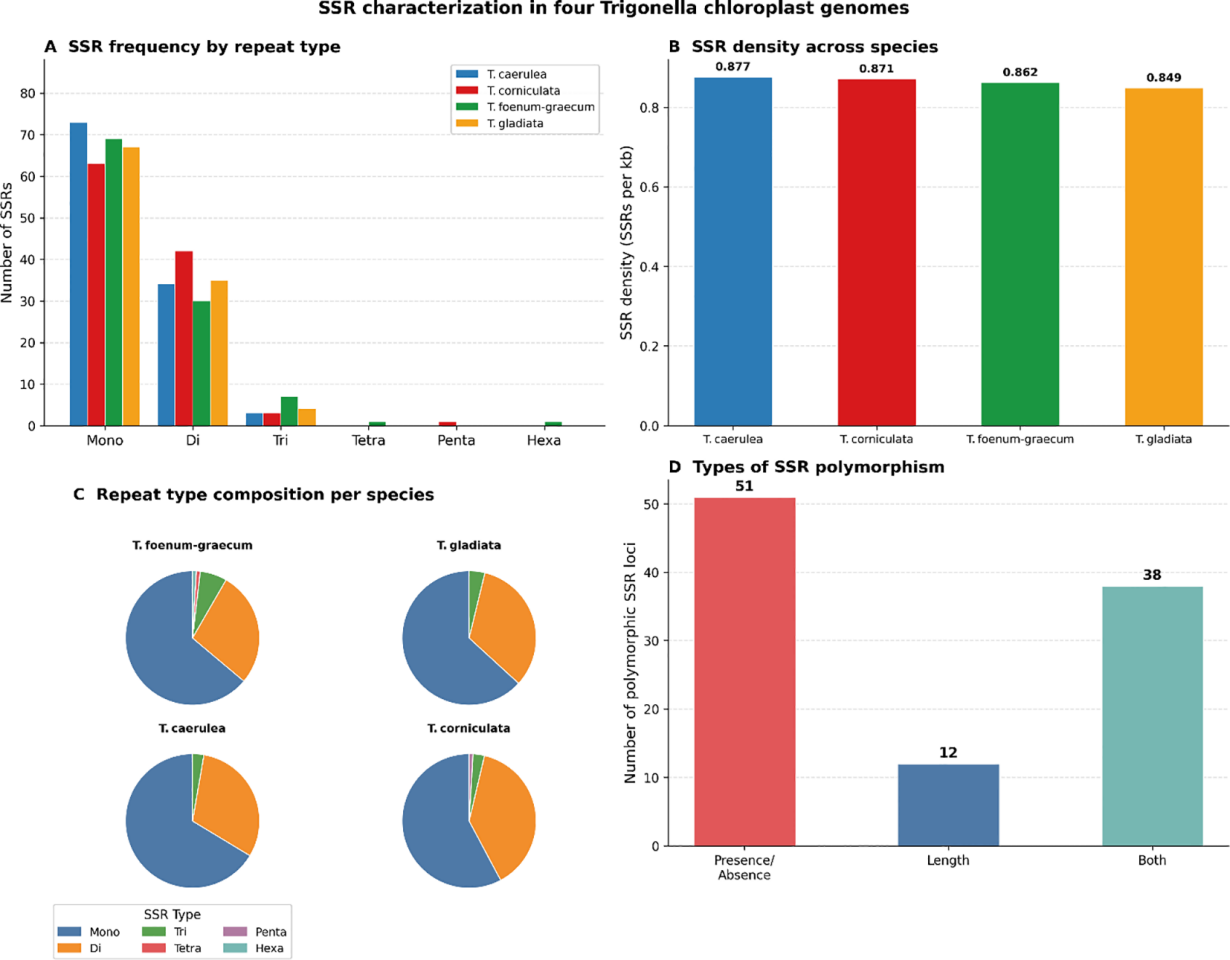
**(A–D)** Characterization of simple sequence repeats (SSRs) in four *Trigonella* chloroplast genomes. **(A)** Frequency of SSRs by repeat type (mononucleotide to hexanucleotide) across the four species, shown as grouped bars. **(B)** SSR density (number of SSRs per kilobase) for each species. **(C)** Proportional composition of repeat types per species shown as pie charts. **(D)** Classification of the 101 polymorphic SSR loci by type of polymorphism: presence/absence only, length only, or both.

SSRs were identified in all four chloroplast genome sequences following minimum repeat thresholds: 10 for mononucleotide, 6 for dinucleotide, 5 for tri- and tetranucleotide, and 4 for penta- and hexanucleotide motifs. A total of 433 SSRs were identified across the four *Trigonella* chloroplast genomes (**Supplementary Tables S2–S5**), ranging from 106 (*T. gladiata*) to 110 (*T. caerulea*), with a consistent density of 0.849–0.877 SSRs/kb (**Figure 5B**; **Supplementary Table 1**). The SSR repertoire was dominated by mononucleotide (57.8–66.4%) and dinucleotide (27.8–38.5%) repeats in all species (**Figures 5A, C**; **Supplementary Tables S2–S5**). At the motif level, the poly-A repeat accounted for 61.9% of all SSRs, followed by AA (21.5%) and AT (10.9%), together comprising over 94% of the total, consistent with the strong AT-compositional bias of plastid genomes (**Supplementary Table 1**).

Comparative analysis identified 101 polymorphic loci (**Figure 5D**; **Supplementary Table 6**), of which 51 showed presence/absence variation only, 12 showed length polymorphism only, and 38 exhibited both simultaneously. A set of 101 primer pairs was developed targeting all polymorphic loci (**Supplementary Table 7**), with mean GC contents of 45.9%/45.7% and mean melting temperatures of 50.1 C/50.0°C for forward and reverse primers, respectively, providing a practical resource for population-level genotyping and species discrimination within *Trigonella* species.

### Selection pressure analysis of protein-coding genes

**Figure 6:**
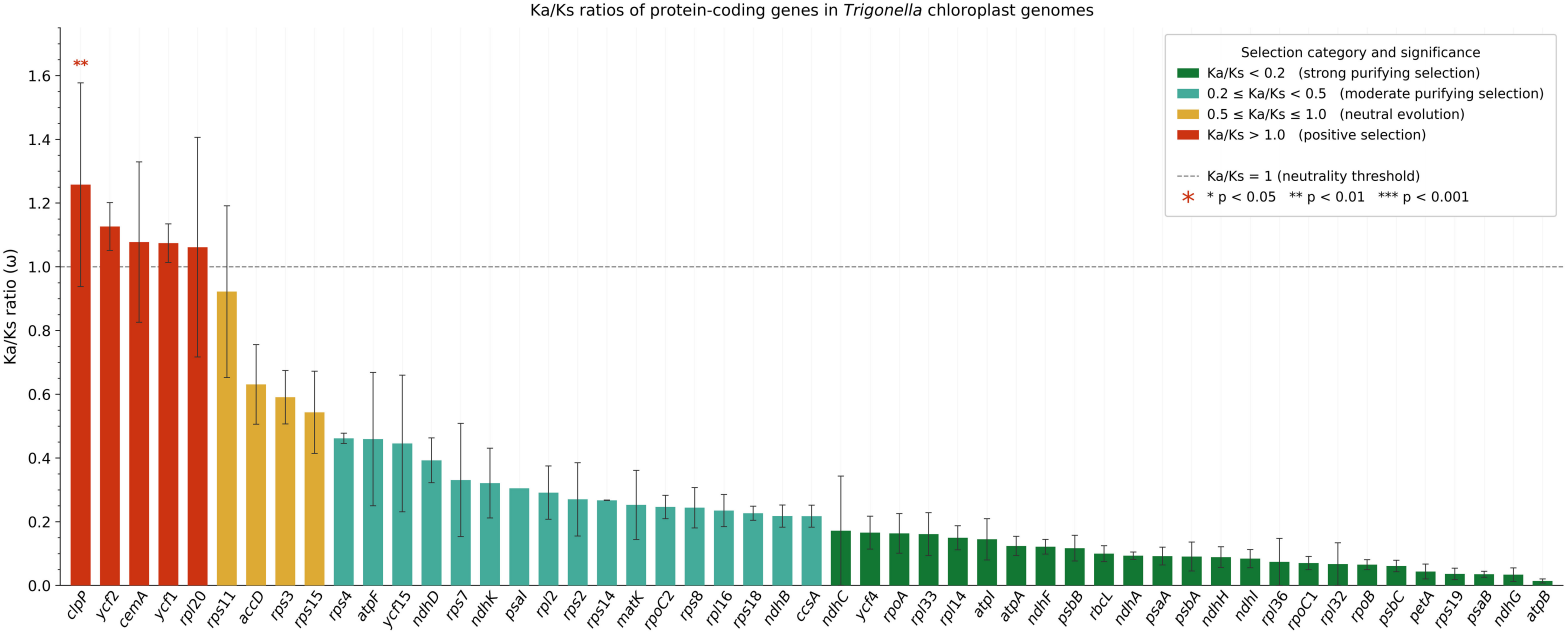
Ka/Ks ratios (ω) of protein-coding genes in *Trigonella* chloroplast genomes. Genes are arranged in descending order of Ka/Ks from left to right. Error bars represent the standard error (SE) of the mean across pairwise comparisons. Bar colors indicate the selection category. The dashed horizontal line marks Ka/Ks = 1, the theoretical boundary between purifying and positive selection. Asterisks above bars denote statistical significance based on Fisher's exact test: * p < 0.05, ** p < 0.01, *** p < 0.001.

To understand the selection pressure, Ka/Ks ratios (ω) were estimated for protein-coding genes shared across all four *Trigonella* species (**Figure 6**). The overwhelming majority of loci evolved under purifying selection: 43 genes (62.3%) had mean Ka/Ks < 0.2, including 18 genes with Ka/Ks = 0, indicating complete amino acid conservation across the genus.

The genes with Ka/Ks = 0 included core photosynthetic subunits (*psbD*, *psbE*, *psbF*, *psbH*, *psbI*, *psbN*, *psbZ*), electron transport components (*petB*, *petD*), ATP synthase subunits (*atpE*, *atpH*), and NADH dehydrogenase subunits (*ndhE*, *ndhJ*). A further 17 genes (24.6%) showed moderate purifying selection (0.2 ≤ Ka/Ks < 0.5), and four genes (5.8%) fell within the neutral range (0.5 ≤ Ka/Ks ≤ 1.0). Five genes exhibited Ka/Ks > 1.0, indicative of positive selection: *clpP* (1.258), *ycf2* (1.126), *cemA* (1.078), *ycf1* (1.074), and *rpl20* (1.062). Only *clpP* reached statistical significance (p = 0.0014), consistent with its documented history of accelerated evolution in angiosperm chloroplast genomes.

### Nucleotide diversity analysis

**Figure 7:**
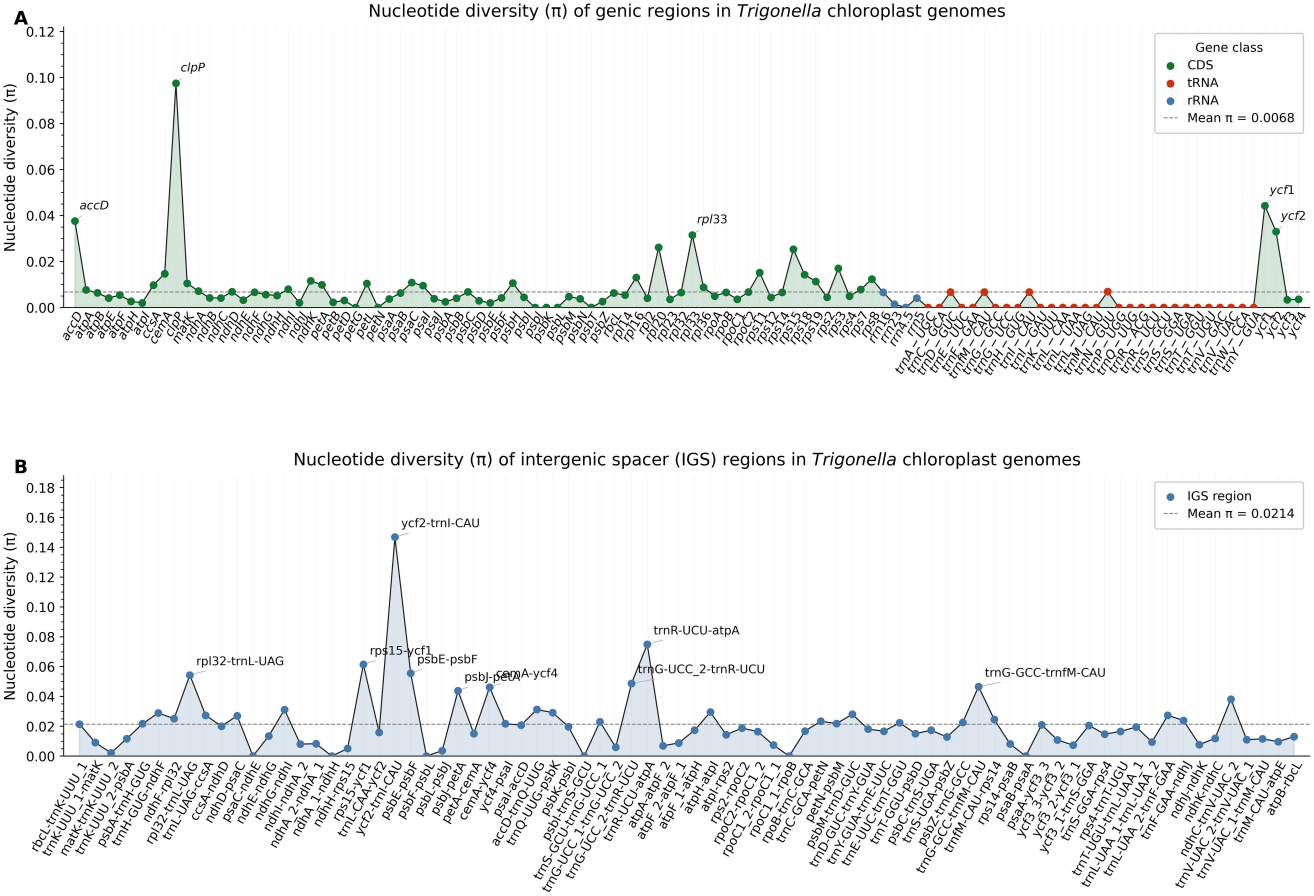
Nucleotide diversity in the chloroplast genome of four *Trigonella* species. **(A)** (genic regions) displays the nucleotide diversity of genes. Markers are color-coded by gene class: green for CDS genes, red for tRNA genes, and blue for rRNA genes. The labelled peaks denote hypervariable genes and intergenic regions. **(B)** (IGS regions) displays the nucleotide diversity of 78 intergenic spacer regions.

Nucleotide diversity (π) was surveyed across 110 genic loci and 78 intergenic spacer (IGS) regions (**Figure 7**). Among genic loci, tRNA and rRNA genes were largely invariant (π = 0), while protein-coding genes showed low to moderate diversity; *clpP* was the most variable CDS (π = 0.0975), consistent with the Ka/Ks > 1 signal recovered in our selective pressure analysis, followed by *ycf1* (π = 0.0443), *accD* (π = 0.0376), and *ycf2* (π = 0.0330), whereas core photosynthetic subunit genes (*psbA*, *psaA*, *psaB*, *rbcL*) were essentially invariant (π < 0.01). IGS regions were generally more variable, with the *ycf2*–*trnI*-CAU spacer exhibiting the highest diversity in the entire dataset (π = 0.1468), followed by *trnR*-UCU–*atpA* (π = 0.0750) and *rps15*–*ycf1* (π = 0.0614), while spacers flanking conserved gene pairs showed zero diversity.

### Codon usage and amino acid composition

Relative synonymous codon usage (RSCU) was analyzed across 77 protein-coding genes in each of the four *Trigonella* plastomes. All four species showed a strong preference for codons ending in adenine (A) or thymine (T), with TTA (Leucine), GCT (Alanine), TCT (Serine), and AGA (Arginine) among the most favored codons (RSCU > 1.5). Codons ending in guanine (G) or cytosine (C) were consistently underrepresented, with TCG, CCG, ACG, GCG, CGC, and CGG falling below an RSCU of 0.5 in all species. Variation between species was negligible (mean SD = 0.015), indicating that codon preferences are essentially identical across the four species (**Supplementary Figure 3**; **Supplementary Table 8**). Leucine was the most abundant amino acid (10.63%), followed by isoleucine (8.98%), serine (7.55%), glycine (6.67%), and phenylalanine (5.88%), a composition that directly reflects the AT-biased codon usage since both leucine and isoleucine are preferentially encoded by AT-rich codons (**Supplementary Figure 4** and **Supplementary Table 9**).

### Phylogenetic analysis

**Figure 8:**
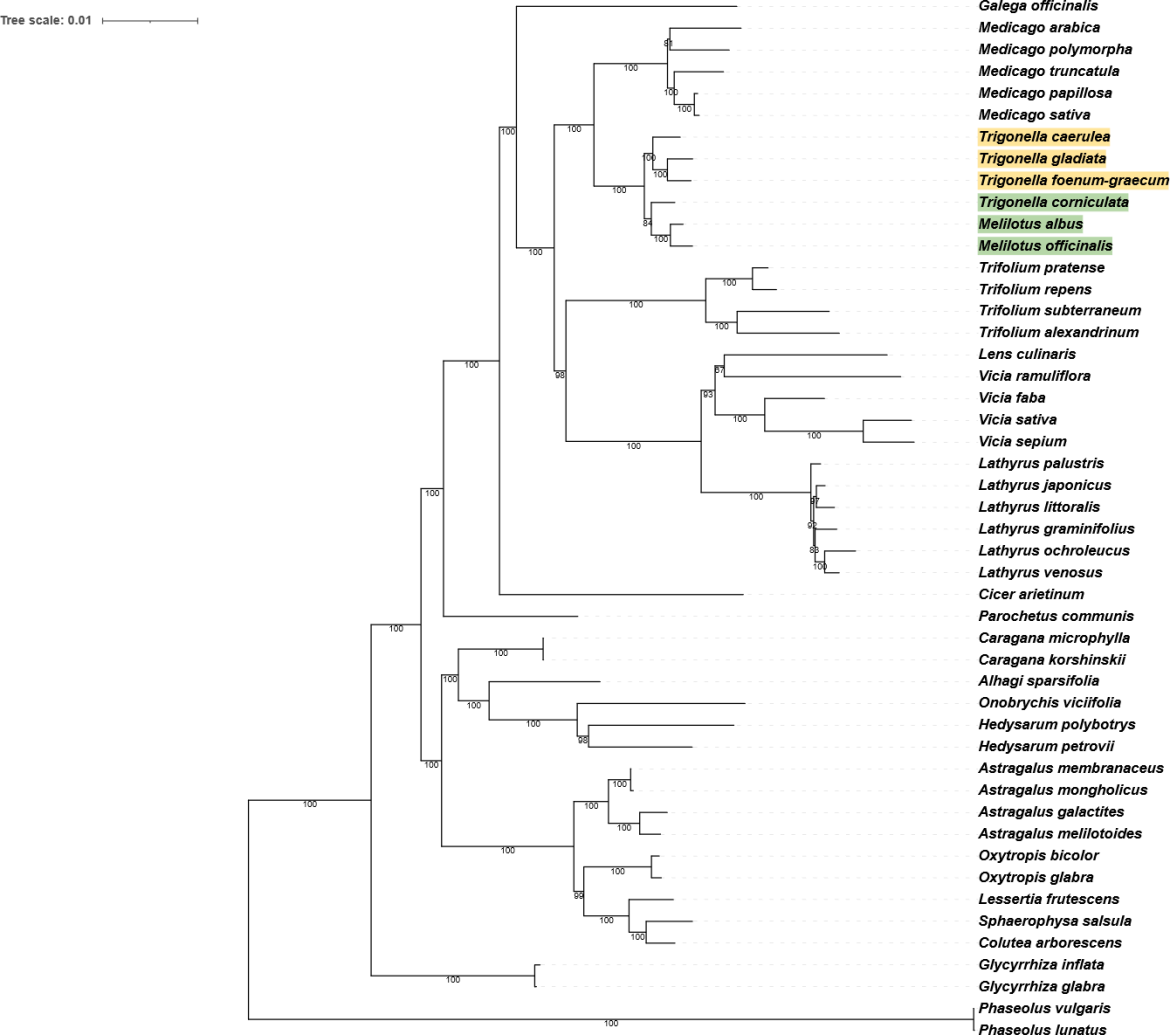
Phylogenetic tree of *Trigonella* along with related taxa in the Papilionoideae subfamily inferred from shared plastid genes. Branch lengths are proportional to the number of substitutions per site (scale bar = 0.01). Numbers at internal nodes represent bootstrap support values (%) derived from 1,000 bootstrap replicates; only values ≥ 50% are shown. The four focal *Trigonella* species (*T. caerulea*, *T. foenum-graecum*, *T. gladiata*, and *T. corniculata*) together with *Melilotus albus* and *M. officinalis* are highlighted. *Phaseolus vulgaris* and *P. lunatus* serve as the outgroup.

To investigate the phylogenetic position of the four *Trigonella* species within the IRLC, we selected 44 taxa from the IRLC clade alongside two outgroup taxa from outside the clade, *Phaseolus vulgaris* and *P. lunatus*. Whole-plastome phylogenetic analysis placed all four *Trigonella* species within the IRLC clade of Papilionoideae subfamily, where they formed a well-supported group together with *Melilotus albus* and *M. officinalis* (bootstrap = 100; **Figure 8**). Within this clade, *T. caerulea*, *T. gladiata*, and *T. foenum-graecum* grouped with full support, while *T. corniculata* was resolved as sister to the two *Melilotus* species rather than to the other *Trigonella* species. This placement confirms the well-documented paraphyly of *Trigonella* with respect to *Melilotus*, with all internal nodes receiving maximum bootstrap support. At a broader scale, the *Trigonella*–*Melilotus* clade was sister to *Medicago*, and together these formed the core Trifolieae alongside *Trifolium*. This Trifolieae assemblage was in turn sister to the *Vicia*-*Lens*–*Lathyrus* alliance, with all groupings receiving full bootstrap support. *Phaseolus vulgaris* and *P. lunatus* served as outgroups at the base of the tree.

## Discussion

In this study, we assembled and annotated the complete chloroplast genomes of four *Trigonella* species, three of which, *T. caerulea*, *T. corniculata*, and *T. gladiata*, have not been reported before. The genome sizes are highly conserved across the four species, ranging from 124,823 bp (*T. gladiata*) to 125,437 bp (*T. caerulea*), consistent with other IRLC legumes such as *Cicer arietinum* (Jansen et al., 2008) and *Lens culinaris* (Tayşi et al., 2022). The GC content (33.71–33.92%) is similarly uniform and reflects the well-documented AT-richness typical of plastid genomes (Daniell et al., 2016; Wolfe et al., 1987). All four genomes encode 77 functional protein-coding genes, 30 tRNA genes, and four rRNA genes, broadly consistent with other IRLC members (Choi et al., 2019).

The gene content of the four species differs in the variable retention of three pseudogenes: *infA*, *rpl22*, and *rps16*. The loss of these genes from the plastid genome is well documented across the IRLC and reflects an ongoing process of endosymbiotic gene transfer (EGT), in which plastid genes are progressively transferred to the nucleus following a copy-then-lose trajectory (Timmis et al., 2004). Nuclear-encoded functional replacements for all three genes have been demonstrated in related legumes, including a notable case in *Medicago truncatula*, where the nuclear copy of *rps16* was recruited from the mitochondrial genome rather than directly from the plastid (Gantt et al., 1991; Millen et al., 2001; Ueda et al., 2008). The successful transfer of these genes requires precise co-evolution between the nuclear-encoded replacement and its plastid-encoded interaction partners, particularly for ribosomal proteins such as L22 and S16, which must be reimported into the chloroplast and integrated into conserved macromolecular complexes (Allen, 2003; Leister, 2003). The gene losses across IRLC members relative to other angiosperms are likely linked to the destabilizing effect of IR loss on plastome integrity (Magee et al., 2010; Wojciechowski et al., 2004).

IR loss also predisposes IRLC plastomes to large-scale structural rearrangements, as the inverted repeat normally suppresses illegitimate recombination by providing substrates for homologous repair (Maréchal and Brisson, 2010; Palmer and Thompson, 1982).

Consistent with this, our whole-genome alignment revealed a complex pattern of structural rearrangements in the chloroplast genome of *T. foenum-graecum*. These rearrangements include four rearranged LCBs, including three inversions and one translocation across an approximately 35.5 kb region. The structural changes identified in *T. foenum-graecum* cv. AFG-3 were independently confirmed by incorporating two publicly available plastome sequences (NC_042857.1 and MN736956.1) into the Mauve alignment, both of which exhibited the same rearrangement pattern relative to the three collinear species. This represents an important addition to the growing catalogue of structural rearrangements documented in IRLC plastomes, including those reported in *Trifolium*, *Medicago*, and *Pisum* (Cai et al., 2008; Magee et al., 2010). The mVISTA alignment further confirmed broadly high sequence identity across all four genomes, with protein-coding exons showing the strongest conservation, reflecting intense purifying selection on the plastid-encoded proteome, while non-coding intergenic regions showed comparatively greater divergence, consistent with their reduced functional constraint.

SSR analysis identified 433 SSRs across the four plastomes, dominated by mononucleotide poly-A repeats, which alone accounted for over 61% of all SSRs. This pattern directly reflects the AT-rich composition of plastid genomes and is consistent with SSR profiles reported across a wide range of angiosperms (Powell et al., 1995; Provan et al., 2001). Comparative analysis identified 101 polymorphic SSR loci, a substantially larger set of markers than has been reported for most individual species within Trifolieae, and the primer pairs developed for these loci provide a practical resource for population-level genotyping, phylogeographic studies, and species discrimination within *Trigonella*.

The selective pressure analysis showed that most chloroplast genes are evolving under strong purifying selection, as expected given their essential roles in photosynthesis and gene expression (Yang et al., 2016). Five genes, *clpP*, *ycf2*, *cemA*, *ycf1*, and *rpl20*, showed Ka/Ks values above 1.0, though only *clpP* reached statistical significance. The elevated evolutionary rate of *clpP* was further supported by the highest nucleotide diversity among coding genes, consistent with its well-documented history of accelerated evolution across angiosperms (Weng et al., 2014; Williams et al., 2019). Nucleotide diversity analysis also identified several highly variable intergenic spacers, including *ycf2*–*trnI*-CAU, *trnR*-UCU–*atpA*, and *rps15*–*ycf1*, which represent strong candidates for molecular marker development across the Trifolieae (Shaw et al., 2007).

Finally, whole-plastome phylogenetic analysis provided the strongest evidence to date for the paraphyly of *Trigonella* with respect to *Melilotus*, with *T. corniculata* resolving as sister to *M. albus* and *M. officinalis* with maximum bootstrap support. This result is consistent with previous studies based on limited plastid and nuclear markers (Dangi et al., 2016; Steele et al., 2010), but with substantially greater resolution afforded by whole-plastome data. The paraphyly of *Trigonella* has been attributed to the use of floral morphological characters, particularly stamen number and pod shape, that appear to have evolved convergently across the Trifolieae, producing a classification that does not reflect true evolutionary relationships (Small, 2011; Steele et al., 2010). Our results strongly support the case for a formal taxonomic revision of *Trigonella* and *Melilotus*, though resolving this question definitively will require broader taxon sampling across both genera.

## Conclusion

This study presents the first comparative chloroplast genome analysis across four *Trigonella* species, *T. foenum-graecum*, *T. corniculata*, *T. caerulea* and *T. gladiata*. Despite broad conservation in genome size, gene content, and nucleotide composition, all consistent with other IRLC legumes, several evolutionarily significant findings emerged. The pseudogene status of *infA*, *rpl22*, and *rps16* across one or more species captures different stages of an ongoing plastid-to-nucleus gene transfer process, reinforcing the view that the IRLC represents a natural experiment in endosymbiotic genome reduction. Whole-genome alignment revealed that the genome organization of *T. foenum-graecum* differs from the three collinear species through a series of inversions and translocations affecting four locally collinear blocks, resulting in reorganized gene order across a ~35.5 kb region. Selective pressure analysis confirmed that purifying selection dominates plastome evolution, while the elevated Ka/Ks and nucleotide diversity of *clpP* mark it as a consistent outlier across angiosperms. Codon usage across all four plastomes is shaped primarily by selection rather than mutational bias, a pattern well documented in photosynthetic plastid genomes. The polymorphic SSR loci and hypervariable intergenic regions identified here provide practical resources for population genetics and phylogeographic studies within the genus. Most critically, whole-plastome phylogenetic analysis provides the strongest evidence to date that *Trigonella*, as currently defined, is not a natural group, with *T. corniculata* resolving as sister to *Melilotus (M. albus* and *M. officinalis)* with maximum bootstrap support.

## Data Availability

The datasets presented in this study can be found in onlinerepositories, at the link https://figshare.com/articles/dataset/Complete_chloroplast_genomes_of_four_i_Trigonella_i_species_i_Trigonella_caerulea_i_i_T_corniculata_i_cv_PKM_i_T_foenum-graecum_i_cv_AGF-3_and_i_T_gladiata_i_/32063721. The names of the repository/repositories and accession number(s) can be found in the article/**Supplementary Material**.
