# Supplementary material for: Comparative chloroplast genome analysis of four Trigonella species: structural rearrangements, gene loss, and phylogenetic relationships
Source: Front Plant Sci. 2026 Jun 11;17:1821377. doi: 10.3389/fpls.2026.1821377 (PMC13294209; doi:10.3389/fpls.2026.1821377)
Supplement: Supplementary file 1 [file Table1.docx]

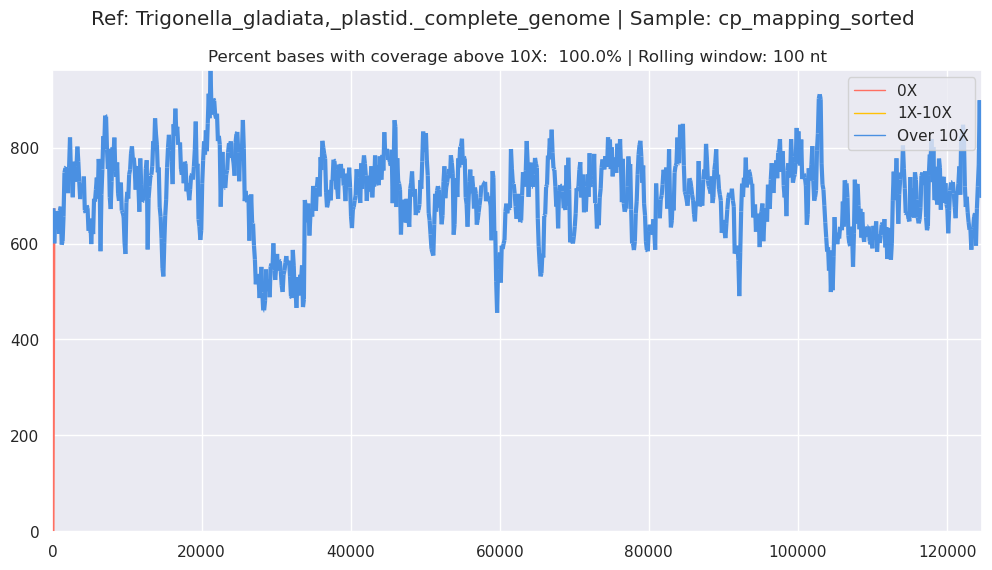


1. ***Trigonella* gladiata**


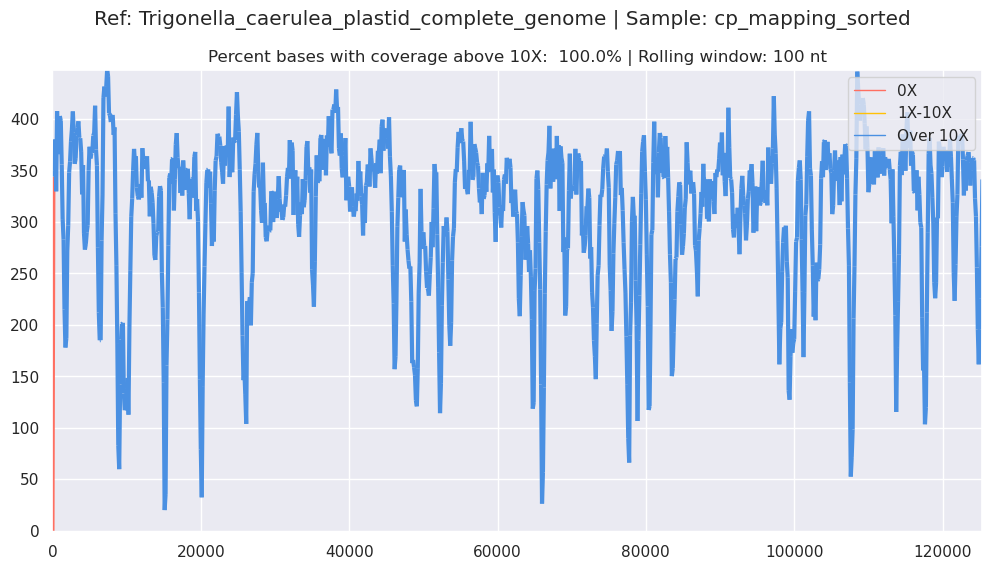


1. ***Trigonella* caerulea**


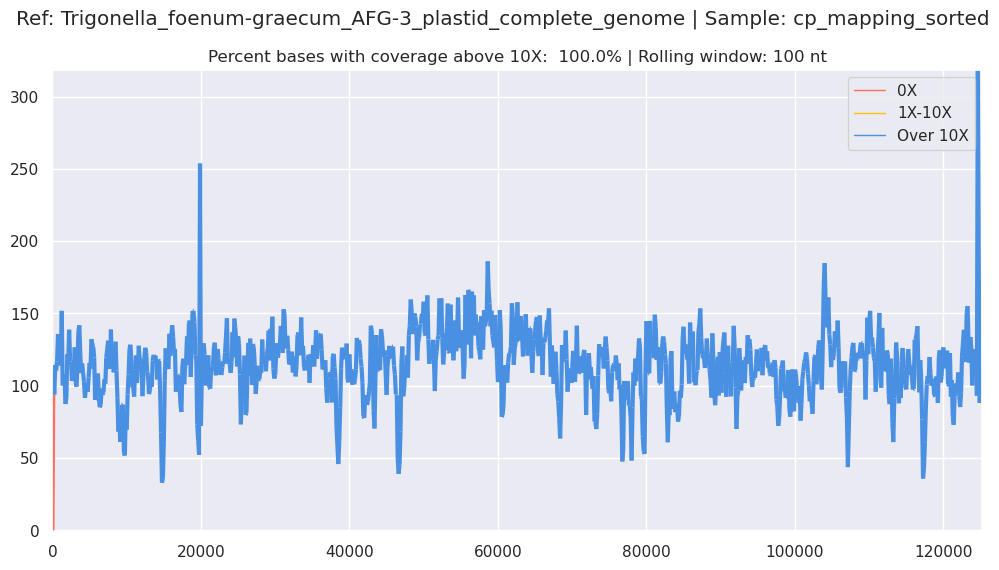


1. ***Trigonella foenum-graecum* cv. AFG-3**


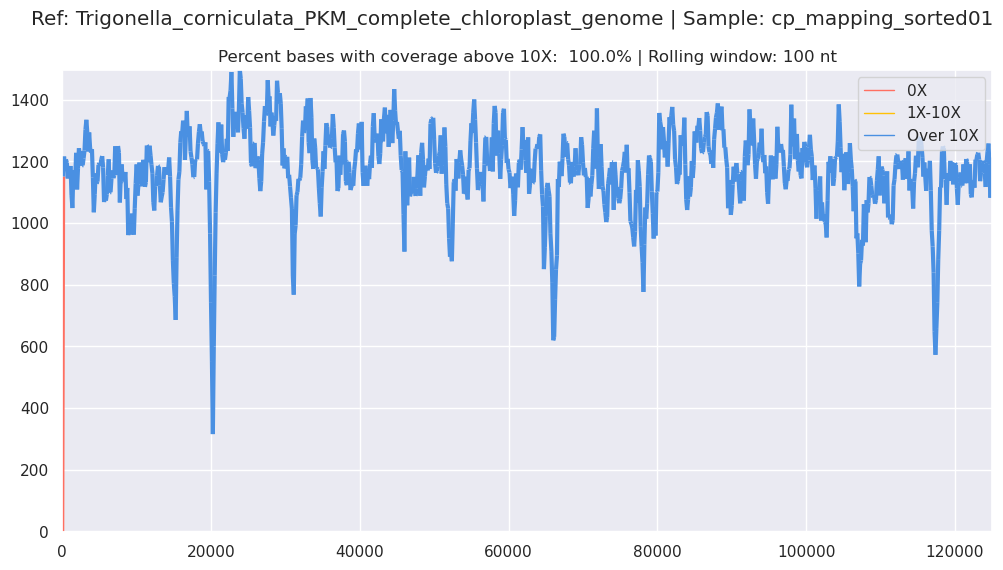


1. ***Trigonella corniculata* cv. Pusa Kasuri Mathi**

**Supplementary Figure S1 A-D.** Read coverage depth across the assembled chloroplast genomes of *Trigonella* species, (A) *Trigonella* *gladiata*, (B) *Trigonella* *caerulea*, (C) *Trigonella foenum-graecum* cv. AFG-3 and (C) *Trigonella corniculata* cv. Pusa Kasuri Mathi. Raw Illumina reads were mapped back to the final assembled genome using BWA-MEM v0.7.19, and coverage depth was plotted using bam2plot. The x-axis represents genomic position (bp) and the y-axis represents sequencing depth (read coverage). Uniformly high coverage across the entire genome length confirms assembly completeness and the absence of major assembly artefacts.


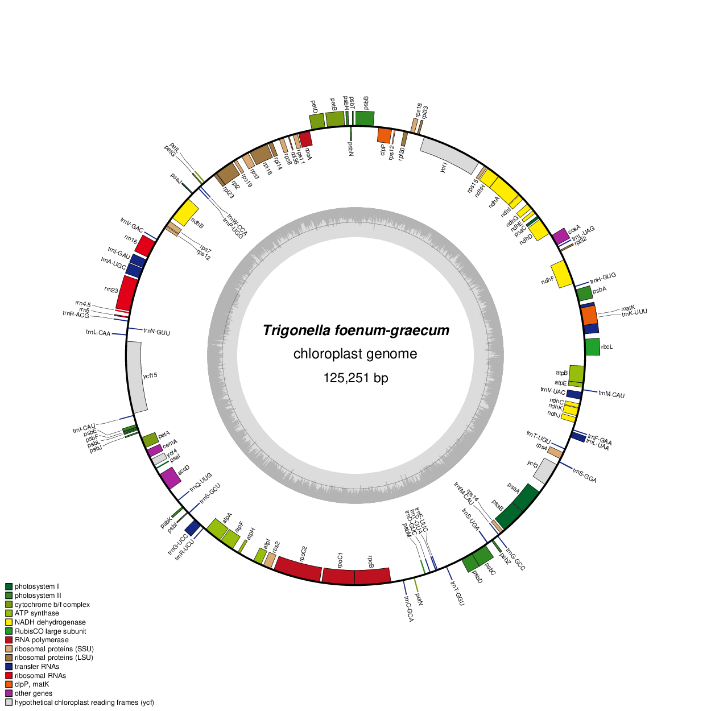


**(A)** *Trigonella foenum-graecum* (125,251 bp).


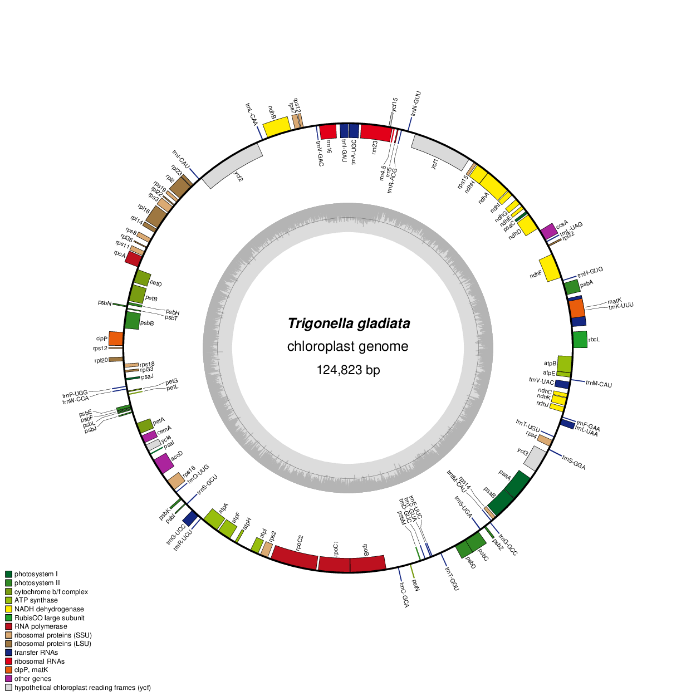


**(B)** *Trigonella gladiata* (124,823 bp).


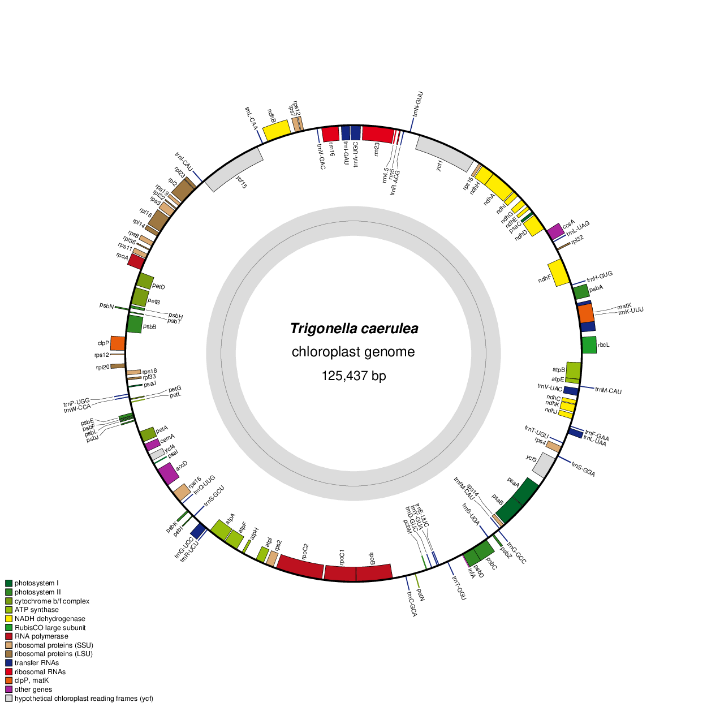


**(C)** *Trigonella caerulea* (125,437 bp).

**Supplementary Figure S2 A-C.** Circular chloroplast genome maps of the three *Trigonella* species, *Trigonella foenum-graecum*, *Trigonella gladiata,* and *Trigonella caerulea*. The circular genome map of T. corniculata cv. Pusa Kasuri Mathi is shown in Figure 1 of the main text. Genes shown on the outer ring are transcribed clockwise; genes on the inner ring are transcribed anticlockwise. The inner grey ring represents GC content across the genome. Gene category is colour coded in each map: dark green, Photosystem I; light green, Photosystem II; teal, cytochrome b6/f complex; yellow, ATP synthase; brown, NADH dehydrogenase; dark yellow, RuBisCO large subunit; dark blue, RNA polymerase; light blue, ribosomal proteins (SSU); purple, ribosomal proteins (LSU); black, transfer RNAs; dark red, ribosomal RNAs; orange, *clpP* and *matK*; light grey, other genes; pale grey, hypothetical chloroplast reading frames (*ycf*).


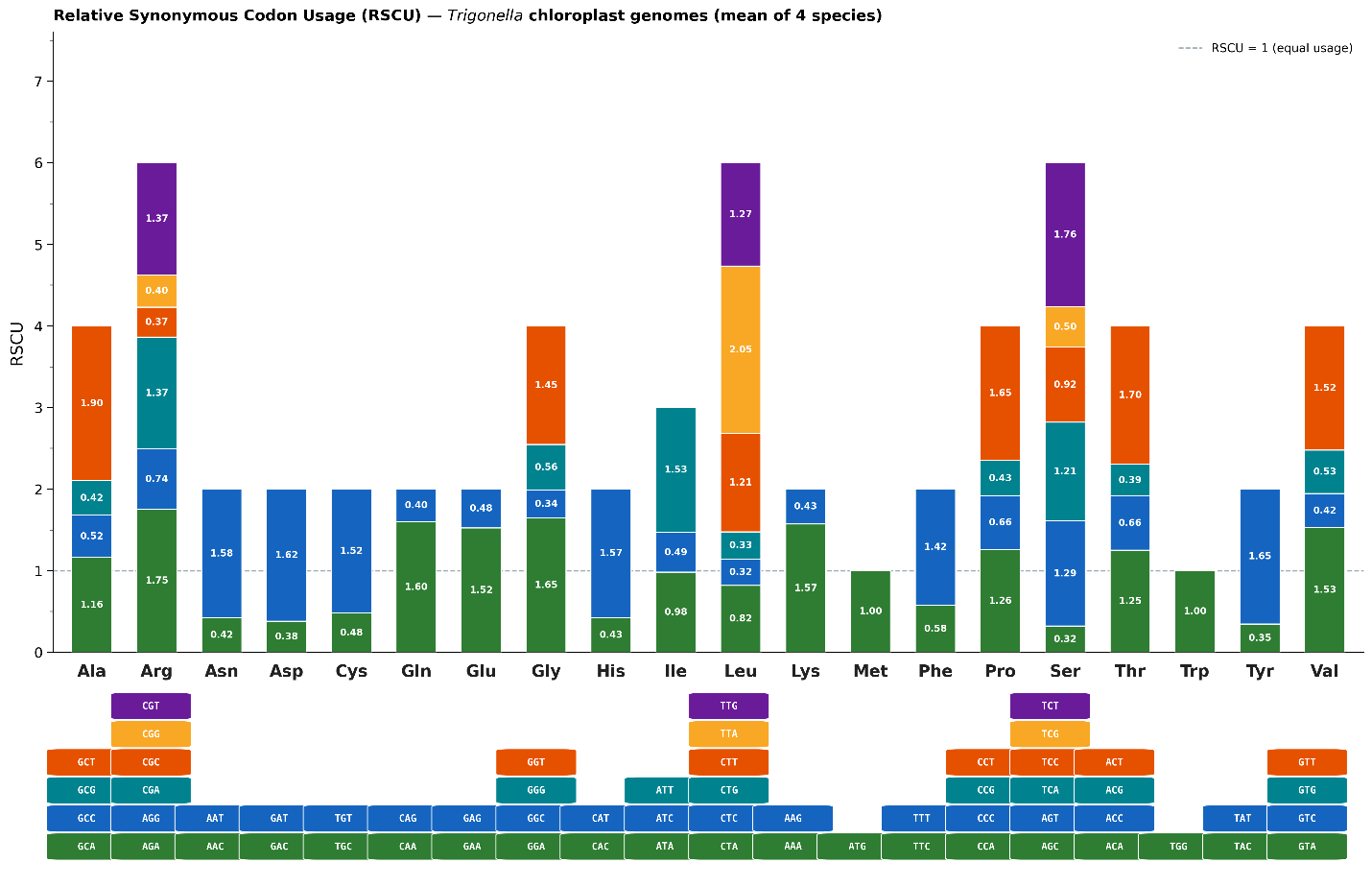


**Supplementary Figure S3.** Relative synonymous codon usage (RSCU) across the four *Trigonella* species chloroplast genomes. Stacked bar chart showing the combined mean RSCU values for all 59 synonymous codons across 77 protein-coding genes in the four *Trigonella* plastomes (*n* = 4 species). Each bar represents one synonymous codon group (amino acid), and the coloured segments within each bar correspond to individual synonymous codons, labelled with their RSCU value. Codons are colour-coded to distinguish synonymous variants within each amino acid group.


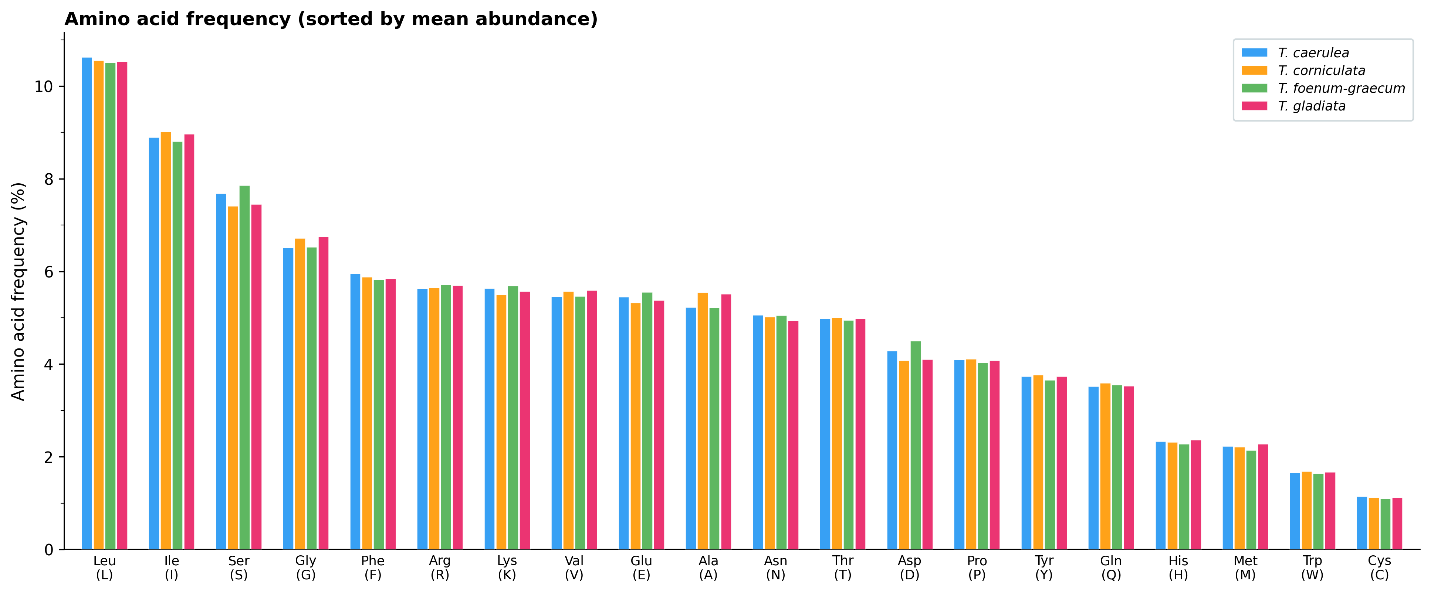


**Supplementary Figure S4.** Amino acid composition of chloroplast-encoded proteins in the four Trigonella species. Grouped bar chart showing the frequency (%) of each of the 20 standard amino acids across the 77 functional protein-coding genes in the four *Trigonella* chloroplast genomes, sorted in descending order of mean abundance. Each species is represented by a different colour: blue, *T. caerulea*; orange, *T. corniculata*; green, *T. foenum-graecum*; pink, *T. gladiata*.
